# Altered neuromagnetic activity under visual stimuli in migraine: a multi-frequency magnetoencephalography study

**DOI:** 10.3389/fneur.2025.1567150

**Published:** 2025-07-30

**Authors:** Di Wu, Zhiyuan Zhou, Yingfan Wang, Hongxing Liu, Yuanwen Yu, Qiqi Chen, Xiaoshan Wang

**Affiliations:** ^1^Department of Neurology, The Affiliated Brain Hospital of Nanjing Medical University, Nanjing, China; ^2^Department of Neurology, Shanghai General Hospital, Shanghai, China; ^3^MEG Center, The Affiliated Brain Hospital of Nanjing Medical University, Nanjing, China

**Keywords:** migraine, magnetoencephalography, multi-frequency, visual stimuli, spectral power, functional connectivity

## Abstract

**Background:**

Migraine is a chronic neurological disorder associated with a variety of abnormal visual symptoms. However, the mechanisms of visual processing in migraine are not fully understood. This study aimed to investigate neuromagnetic activity abnormalities under visual stimuli in migraine patients using magnetoencephalography (MEG).

**Methods:**

Magnetoencephalography recordings during exposure to visual stimuli were collected for 22 episodic migraine patients without aura during the interictal period and 22 age- and gender-matched healthy controls (HCs). The spectral power and functional connectivity (FC) of visual neuromagnetic activation were estimated using minimum norm estimation combined with the Welch technique and corrected amplitude envelope correlation.

**Results:**

Compared to HCs, migraineurs exhibited attenuated spectral power in the gamma2 band of the bilateral medial orbitofrontal cortices, bilateral posterior cingulate cortices, bilateral temporal poles, right lateral orbitofrontal cortex, and left transverse temporal cortex. Migraineurs also exhibited the following increases in FC relative to HCs between vision- and pain-related brain regions: In the alpha band, FC between the left cuneus and left medial orbitofrontal cortex was significantly increased. In the beta band, FC between the left lateral occipital cortex and bilateral anterior cingulate cortices was significantly increased. In the gamma1 band, FC of the right cuneus with the bilateral insulae, left parahippocampal cortex, bilateral posterior central cortices, and bilateral anterior cingulate cortices was significantly increased. Migraineurs also showed significantly increased FC between the left lateral occipital cortex and the right medial orbitofrontal cortex, left posterior central cortex, and bilateral anterior cingulate cortices. The clinical variables (headache history, attack frequency, and pain intensity) had no significant correlation with MEG results.

**Conclusion:**

Taken together, these findings demonstrate altered spectral power in pain-processing regions and altered FC between vision and pain-related regions in migraineurs under visual stimuli in multi-frequencies. These results may contribute to understanding the relationship between visual dysfunction and headache onset in migraineurs, providing valuable insights into the underlying pathophysiology.

## Introduction

Migraine is a chronic neurological disorder characterized by moderate or severe headache attacks accompanied by photophobia, phonophobia, cutaneous allodynia, and gastrointestinal symptoms such as nausea and emesis (1). Among these accompanying symptoms, photophobia is one of the main discriminating symptoms between migraine and other headache disorders (2). Migraineurs report a variety of abnormal visual symptoms besides photophobia. Visual aura occurs in over 90% of patients with aura (3), which is believed to arise from cortical spreading depression (CSD) beginning from the occipital cortex (4). Furthermore, approximately 70% of migraine patients experience non-aura visual symptoms, such as palinopsia and visual snow (1, 2), while 40% report that visual stimuli can trigger migraines (5). Taken together, these findings suggest that the migraine patients may have abnormal processing of visual information during and between the headache attacks.

In recent years, many neuroimaging studies have demonstrated that migraine patients have abnormal processing of visual information. Most magnetoencephalography (MEG) studies have shown that repetitive visual stimulus presentation significantly increased the P100m response amplitude, and other studies have found that direct current (DC) shifts were observed in the ictal migraine with aura. Both P100m responses and DC shifts were mainly localized to the primary visual cortex (6). Electroencephalography (EEG) studies have found that migraine patients have alpha defects in the prophase of visual stimulation and increased occipital responsiveness to visual stimulation, supporting the notion that migraine patients have a hyperresponsive visual cortex (7, 8). Functional magnetic resonance imaging (fMRI) studies have shown that migraine patients with aura viewing visually stimulating patterns show greater activation of the primary and extrastriate visual cortices (9, 10). Although these studies suggest that migraine patients show abnormal activation in response to visual stimuli mainly in vision-associated brain areas, other studies have reported hyperresponsiveness of the visual cortex beyond early visual areas. A MEG study have reported that visual neuromagnetic activation in the 5–300 Hz range were observed beyond the visual cortex, especially in the parietal and temporal regions (11). An EEG study have reported that interictal relative theta power was increased in migraine in the frontocentral, parieto-occipital, and temporal region compared to controls under visual stimulation (12). A fMRI study demonstrated that migraineurs show abnormal modulation in response to visual motion stimuli within the visual cortex (superior and inferior occipital gyrus), as well as within the middle frontal gyrus, pontine nuclei, and cerebellar lobules (13). As functional imaging studies have not yet reached a consensus on the relevant changes in brain activation under visual stimulation, more research methods are needed to further explore abnormalities in vision-associated brain activation in migraine patients. MEG is a non-invasive neuroimaging technique that directly captures electrophysiological activity throughout the entire brain. Although MEG matches EEG in millisecond-level temporal resolution and spectral discrimination capabilities, MEG can locate the origins of underlying neural sources with superior spatial resolution compared to EEG (6).

Functional connectivity (FC) is used to evaluate the temporal correlation of spatially distant neurons (14), which can effectively describe collaborative working patterns between brain regions. Connectivity research indicates migraine involves a widespread interictal network disorder, extending beyond isolated dysfunction in primary or secondary sensory areas (15). There is evidence that migraineurs have abnormal visual FC networks. A resting-state MEG study has shown that the migraine with aura had significantly increased FC in the bilateral occipital areas in the theta band, suggesting a key role for the visual cortex in migraine pathophysiology (16).‌ An EEG study has found that migraine patients showed low spatial coherence of alpha activity during visual stimulation (17). A study using resting-state fMRI to study changes in regional visual cortex FC using regional homogeneity (ReHo) and amplitude of low frequency fluctuations (ALFF) found decreased ReHo and ALFF values in the right lingual gyrus, but increased ALFF values in the prefrontal cortex, in migraine patients (18). However, most of these studies were conducted in a resting-state and other studies were performed during visual stimulation. The intrinsic resting-state visual FC network is thought to differ from the network induced by visual stimuli. Although these two networks are interconnected, the latter reflects the unique information processing network of visual stimuli (19). Few studies use MEG to explore the changes in the FC network of migraine patients under visual stimulation. Whether alterations in magnetic signals of the visual connectivity network exist in migraine patients remains unclear. Meanwhile, although there have been studies on visual FC networks, few studies focused on FC changes between vision- and pain-related brain regions in migraineurs. One study showed that migraineurs exposed to light show greater activation of the visual cortex compared to controls, and that this effect is enhanced during thermal painful stimulation of the face (20). A randomized, double-blind clinical study demonstrated that specially designed optical tints, which minimize activation of photosensitive retinal ganglion cells, could potentially alleviate both migraine-related pain and photophobia symptoms (21). These studies suggest an interrelationship between visual functional abnormalities and pain in migraineurs. Therefore, studies on the FC changes between pain and visual processing brain regions in migraine patients may provide a new perspective for elucidating the relationship between abnormal visual processing processes and migraine attacks. Hence, in this study we aimed to explore the FC changes between vision- and pain-related brain regions in patients with migraine. We selected eight vision-related regions of interest (ROIs), including the bilateral cuneus, occipital cortex, lingual cortex, and calcarine cortex, and 14 pain-related ROIs, including the bilateral insula, lateral orbitofrontal cortex, medial orbitofrontal cortex, parahippocampal cortex, posterior central cortex, posterior cingulate cortex, and anterior cingulate cortex, for FC analysis. According to previous vision studies, the cuneus is involved in visual selective attention (22), the occipital cortex is involved in primary processing of visual information (23), and the calcarine cortex and lingual cortex are involved in phosphene perception (24), visual processing, and spatial memory (25). Most of these brain regions are key nodes in the visual network and have been reported to show abnormalities in prior visual stimuli-induced brain activation studies of migraine patients (26). According to previous studies of pain-related brain areas, the posterior insula and posterior central cortex are involved in sensory-discriminative processing of pain (27), the anterior insula and anterior cingulate cortex are involved in affective emotional processing (28, 29), the parahippocampal cortex and orbitofrontal cortex are involved in cognitive processing (28, 30), and the posterior cingulate cortex is involved in nociception and the development of chronic pain (31). Most of these brain regions are key parts of the pain matrix (32) and are the most common aberrant brain regions in previous FC studies of migraine (33).

Based on this background, the present study aimed to investigate spectral and FC changes in response to visual stimuli in migraineurs using MEG in the low to high frequency ranges. Firstly, at the spectral power level, we explored changes in spectral power in migraineurs under visual stimuli compared to healthy controls (HCs). Secondly, at the FC level, we investigated whether migraineurs demonstrate abnormal connectivity between vision- and pain-related brain regions in distinct frequency ranges. Finally, we evaluated whether abnormal spectral power and FC are correlated with clinical characteristics in migraineurs.

## Methods

### Ethics statement

This study was approved by the medical ethics committee of Nanjing Brain Hospital. All participants provided written informed consent form.

### Participants

The inclusion criteria for patients with episodic migraine without aura were based on the International Classification of Headache Disorders, 3rd Edition beta version (ICHD-III beta) (34). The exclusion criteria were: (1) existence of other neurological diseases and (2) the use of prescription medications within 1 week prior to the study. HCs were recruited to match migraine group participants in terms of age and gender. Inclusion criteria for HCs were: (1) no history of any neurologic disorder and (2) no first-degree relative with a history of any type of migraine. The exclusion criteria for all participants were: (1) presence of an implant (e.g., braces, pacemaker) that may result in visible magnetic noise in MEG data; (2) demonstration or expression of noticeable anxiety and/or inability to readily communicate with MEG operators; (3) inability to keep still during scanning; (4) currently pregnant; or (5) claustrophobic tendencies (due to MRI scan).

A total of 22 migraine patients (20 females; mean age: 34.6 years, standard deviation (SD): 6.6 years) were recruited from Nanjing Brain Hospital (Table 1). In addition, 22 age- and gender-matched HCs (20 females; mean age: 33.3 years, SD: 6.8 years) were recruited (Table 1). All participants were right-handed.

**Table 1:** Demographic and clinical characteristics of subjects.

Parameters	Migraine	Control
Gender (female/male)	20/2	20/2
Age (years) (mean ± SD)	34.6 ± 6.6	33.3 ± 6.8
Handedness (left/right)	0/22	0/22
Years of migraine (mean ± SD)	8.6 ± 5.7	NA
Frequency of headache per month (mean ± SD)	2.1 ± 1.4	NA
Durations of migraine attacks (hours)	23.5 ± 27.8	NA
Locus of headache (unilateral/bilateral)	12/10	NA
Pain type (number of subjects)		
Throbbing	15	NA
Pressure	1	NA
Constant	3	NA
Sharp	1	NA
Squeezing	1	NA
Stabbing	1	NA
Severity of headache (VAS scale)	6.4 ± 1.5	NA
Accompanied symptoms with attack (number of subjects)		
Photophobia	13	NA
Phonophobia	13	NA
Nausea/Vomiting	11	NA
Prophylactic treatments in recent 3 months (yes/no)	0/22	NA

SD, standard deviation; N/A, not available; VAS, visual analog scale.

The recruited migraine patients experienced no migraine headache attacks during MEG recording and were headache free for at least 72 h prior to testing and 24 h after scanning. Migraine patients’ clinical characteristics were recorded prior to MEG using a clinical questionnaire including: headache history, headache frequency, duration of headache attacks in the last month, headache locus, headache type, pain intensity assessed by the Visual Analogue Scale (VAS), and accompanying symptoms (e.g., phonophobia, photophobia, nausea, vomiting).

### Stimuli and procedure

During MEG scans, all participants were instructed to stare at a fixed yellow dot in the middle of a checkboard pattern on a screen located approximately 32 cm in front of them. Participants were asked to try to limit blinking, as this may cause noise in MEG data. Pattern-reversal checkerboard stimuli were generated using Brain X customized software (Jing Xiang, Ohio, United States, Cincinnati Children’s Hospital) (35). The visual stimulus consisted of three consecutive reversal patterns with a reversal rate of 1 Hz. These patterns were sequentially presented as full-field, left-field, and right-field. Each pattern was displayed for 600 ms, with a 400 ms gap between patterns. The size of the checkboard was 60 min of arc, which extended 15 (width) × 22 (height) in the left hemifield of the participant with the average luminance set at 12 cd/m^2^ with contrast of 0.94. There was a 400 ms delay between the start of the trigger and the presentation of the stimulus on the screen, which was subtracted from the target time window. The visual task stimulus consisted of 100 triggers for each field type (left, right, and full field) for one set of recordings (11). For this study, we used full-field data for MEG analysis. The stimulus presentation and visual neuromagnetic signal recordings were performed with Brain X software, with the 100 responses automatically collected and averaged by the software. Each set lasted approximately 5 min and each participant completed two sets.

### MEG recording

The MEG signals were recorded in a magnetically-shielded room using a whole-head CTF 275-channel MEG system (VSM MedTech Systems, Inc., Coquitlam, BC, Canada) at the MEG Center at Nanjing Brain Hospital. Before data acquisition, participants were asked to remove all metal from their body. Electromagnetic coils were attached to the nasion and left and right preauricular points of each participant. These three coils were subsequently activated at different frequencies to measure head position relative to the MEG sensors. To capture sensor and background noise, a 3-min empty room recording was performed prior to the MEG data recording and used to calculate the noise covariance for source analysis. The MEG sample frequency was 6,000 Hz. Throughout the scanning process, participants were instructed to lie in a comfortable position with their arms resting at their side. The acquisition window was set at 1000 ms for each trial, and the 400 ms after the trigger (presence of the reversal pattern) was recorded by the MEG system. MEG data were recorded after a third-order gradient noise cancelation process. Head position was measured at the beginning and end of scanning. If head movement during one scan exceeded 5 mm, the dataset was considered “bad” and an additional dataset was recorded.

### MRI scanning

The MRI scans were conducted using 1.5 T MRI (Singa, GE, United States). Three fiducial points were placed at the same locations to facilitate co-registration of MEG and MRI data; these points were regarded as the positions of the three coils used in the MEG recordings before the MRI scan. Subsequently, all anatomical landmarks digitized in the MEG scan were identified during MRI scanning.

### Data preprocessing

A published software, Brainstorm, was used to process MEG data, which is available for free download under the GNU General Public License (36). The following strategies were used to eliminate signals from non-brain activity and environmental artifacts from MEG data: (1) All data were visually inspected. In the case of significant head position bias or artifactual segments caused by noise interference, the contaminated segments were eliminated. (2) Power line contamination was eliminated utilizing a notch filter (50 Hz and its harmonics). (3) MEG recordings began with a 3 min empty-room recording to capture environment and sensor noise, which was used to calculate the noise covariance for source analysis to account for remaining and stationary instrumental, sensor, and environmental noise components. Principal components that met the artifact sensor topology were hand-picked and excluded utilizing orthogonal projection (37). T1-weighted structural volume images were automatically reconstructed in the surface model using the FreeSurfer image analysis package for source investigation^1^. Topographical 3D descriptions of the brain surface generated using integrated geometric reconstructions of the scalp, brain gray matter, and brain white matter were utilized to estimate gray and white matter boundaries. We chose the following frequency bands for MEG data analysis: theta (5–7 Hz), alpha (8–12 Hz), beta (15–29 Hz), gamma1 (30–59 Hz), and gamma2 (60–90 Hz). Neuromagnetic signals < 5 Hz were not included in the analysis because a longer time window is required for the computation of low-frequency components of the data. The present study focused on the time domain of 0–200 ms following the visual trigger, which includes all visual neuromagnetic signal. Selection of this time domain was based on our previous work (11).

### Spectral power analysis of visual stimulation MEG data

Depth-weighted minimum norm estimation (MNE) was utilized to estimate source-level-based cortical activation from the MEG data. Several studies have confirmed the robustness of the MNE approach (38, 39). A forward model of the MNE analysis was constructed using the overlapping sphere method, whereby each cortical vertex was represented as a current dipole. The model comprised around 15,000 vertices. Next, an inverse operator was computed to estimate the current source distribution of the sensor recording data using the following procedure: (1) the direction of the source was restricted to be perpendicular to the surface of the cortex. (2) A depth-weighted algorithm was employed to compensate for biases that affect the calculation of superficial sources. (3) A regularization of value λ2 = 0.33 was used to reduce numerical instability, reduce the noise sensitivity of the MNE, and produce a spatially smooth solution. The regularization parameter determines the weight of the MEG signal model relative to the background noise model, which is defined as the inverse of the signal-to-noise ratio (SNR) of the MEG record. The default SNR in Brainstorm software is “3,” which adopts the definition of SNR in the original MNE software (40).

The cortex of the whole brain was parcellated and aligned into 68 distinct ROIs with structures in the Desikan-Killiany cortical atlas using FreeSurfer 7.1.1 software. In the Desikan-Killiany atlas, 34 cerebral ROIs are present per hemisphere (41). The relative current powers of all vertices in the ROIs were calculated to estimate the source-dependent oscillatory power. The power spectral density (PSD) of each ROI was calculated using the Welch technique (5 s window duration; 50% overlap) (36, 42). The PSD values denote the spectral power of each participant. The spectral power values at every frequency band were modified proportionally to the total power across the entire spectrum as follows:


$$\text{Relative}\;\mathrm{PSD}(f)=\mathrm{PSD}(f)/\sum_{i}[\text{Total}\;\mathrm{PSD}(f_{i}\;)]$$

Where f_i_ represents the individual frequency band from the original PSD. The numerator of the formula represents the initial PSD value of the present frequency band, while the denominator signifies the total of all chosen frequency bands’ original PSD figures. The relative PSD value ranges from 0 to 1, illustrating the contribution of the current frequency band to the total signal power. This method normalizes spectral power across both brain regions and all participants, enhancing inter-individual comparability (42).

### FC analysis of visual stimulation MEG data

For the FC analysis, we selected eight vision-related ROIs, including the bilateral cuneus, occipital cortex, lingual cortex, and calcarine cortex, and 14 pain-related ROIs, including the bilateral insula, lateral orbitofrontal cortex, medial orbitofrontal cortex, parahippocampal cortex, posterior central cortex, posterior cingulate cortex, and anterior cingulate cortex, using the Desikan-Killiany atlas. To calculate FC between vision- and pain-related brain regions, corrected amplitude envelope correlation (AEC-c) analysis was utilized to estimate oscillatory FC between ROIs. Previous research has shown that AEC-c analysis offers high levels of repeatability and stability in FC network research (43). Before computing the signal envelopes to remove spurious connections caused by volume conduction effects and field spread, we orthogonally aligned the signal pairs using a previously described method (44). The amplitude envelope was defined as the absolute value of the Hilbert transform of a particular cortical oscillation, acquired through band-pass filtering of cortical source activity for each frequency band, which indicates the fluctuation of amplitude over time (45). If s(t) is an arbitrary time series, the Hilbert transform can be defined as:


$$s_{H}(t)=\frac{1}{\pi }\int_{-\infty }^{+\infty }\frac{s(\tau )}{\left(t-\tau \right)}\mathrm{d\tau}$$

Where SH(t) represents the input real-valued signal (a time-domain function), *τ* serves as the integration dummy variable indicating time offset, and t specifies the target time point at which the output signal value is evaluated. The Hilbert envelope was divided into *n* equal-length time periods. Subsequently, the average value of the envelope was computed for each time window. Pearson correlation coefficients were calculated for the average envelopes to measure FC, which is reflected by the AEC-c value. The AEC-c values were derived by correlating the amplitude envelopes of cortical oscillatory activity between two ROIs. A high AEC-c value indicates that the amplitude envelope has strong synchronized fluctuations between the two cortical ROIs, reflecting strong FC (46, 47). Finally, the AEC-c values for all participants for all selected ROIs were calculated and the full 8 × 14 adjacency matrix was estimated.

### Statistical analysis

SPSS version 27.0 (IBM, Inc.) software was used for statistical analysis. The Kolmogorov–Smirnov test was used to assess the data distribution. Since the data of spectral power and FC did not conform to normal distribution, the Mann–Whitney test was then used to compare the spectral power for each ROI in each frequency band between the two groups (migraineurs and HCs), as well as group differences in AEC-c values that represent the FC strength between ROI nodes. The correlations between migraine clinical variables (headache history, attack frequency, and pain intensity) and abnormal MEG results (spectral power and FC) were analyzed using Spearman correlation coefficients. The threshold of statistical significance was set at *p* < 0.05, with false discovery rate (FDR) correction. FDR correction was implemented using the Benjamini and Hochberg method (proposed in 1995) via the statistical analysis website^2^.

## Results

### Clinical characteristics

All 22 migraine patients (100%) suffered from migraine without aura. Of these patients, 20 (91%) were females, 21 (95%) presented moderate to severe headache, and 12 (55%) manifested unilateral headache attacks (Table 1).

### Spectral power analysis

Differences in spectral power under visual stimuli between the migraine and HC groups were largely concentrated in the gamma bands. There were no significant group differences in spectral power of the theta, alpha, or beta frequency bands.

**Figure 1:**
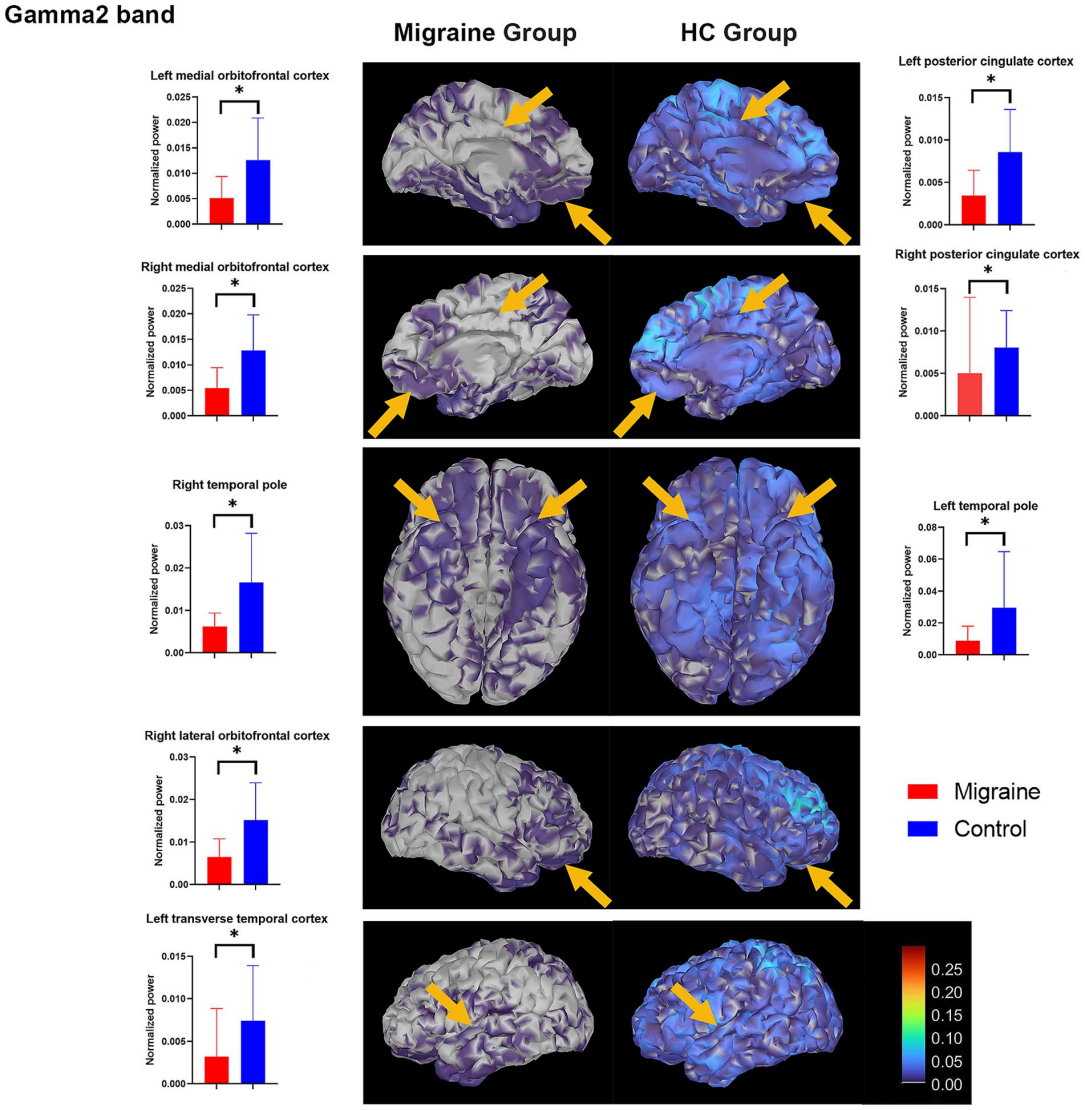
Significant differences in spectral power between the migraine and HC groups. The spectral power maps are shown from the lateral and bottom views. The current power of the underlying cortical sources is color-coded, with larger values represented in red and smaller values in blue. In the gamma2 band, the migraine group had significantly lower spectral power in the bilateral medial orbitofrontal cortices, bilateral posterior cingulate cortices, bilateral temporal poles, right lateral orbitofrontal cortex, and left transverse temporal cortex (indicated by yellow arrows). *p-values after correction were less than 0.05.

In the gamma2 (60–90 Hz) band, the migraine group had significantly lower spectral power in the left medial orbitofrontal cortex (*p_corrected_* = 0.009), right medial orbitofrontal cortex (*p_corrected_* = 0.009), left posterior cingulate cortex (*p_corrected_* = 0.009), right posterior cingulate cortex (*p_corrected_* = 0.009), left temporal pole (*p_corrected_* = 0.009), right temporal pole (*p_corrected_* = 0.009), right lateral orbitofrontal cortex (*p_corrected_* = 0.009), and left transverse temporal cortex (*p_corrected_* = 0.009) compared with the HC group (Figure 1). These brain regions showed a trend of decreased spectral power in the gamma1 band (30–59 Hz), but the difference was not statistically significant.

### FC analysis

The FC between vision- and pain-related brain regions under visual stimuli showed specific alterations across the frequency bands. Differences in FC between the migraine and HC groups were mainly concentrated within the alpha, beta, and gamma1 frequency bands. There were no significant group differences in the theta or gamma2 frequency bands.

**Figure 2:**
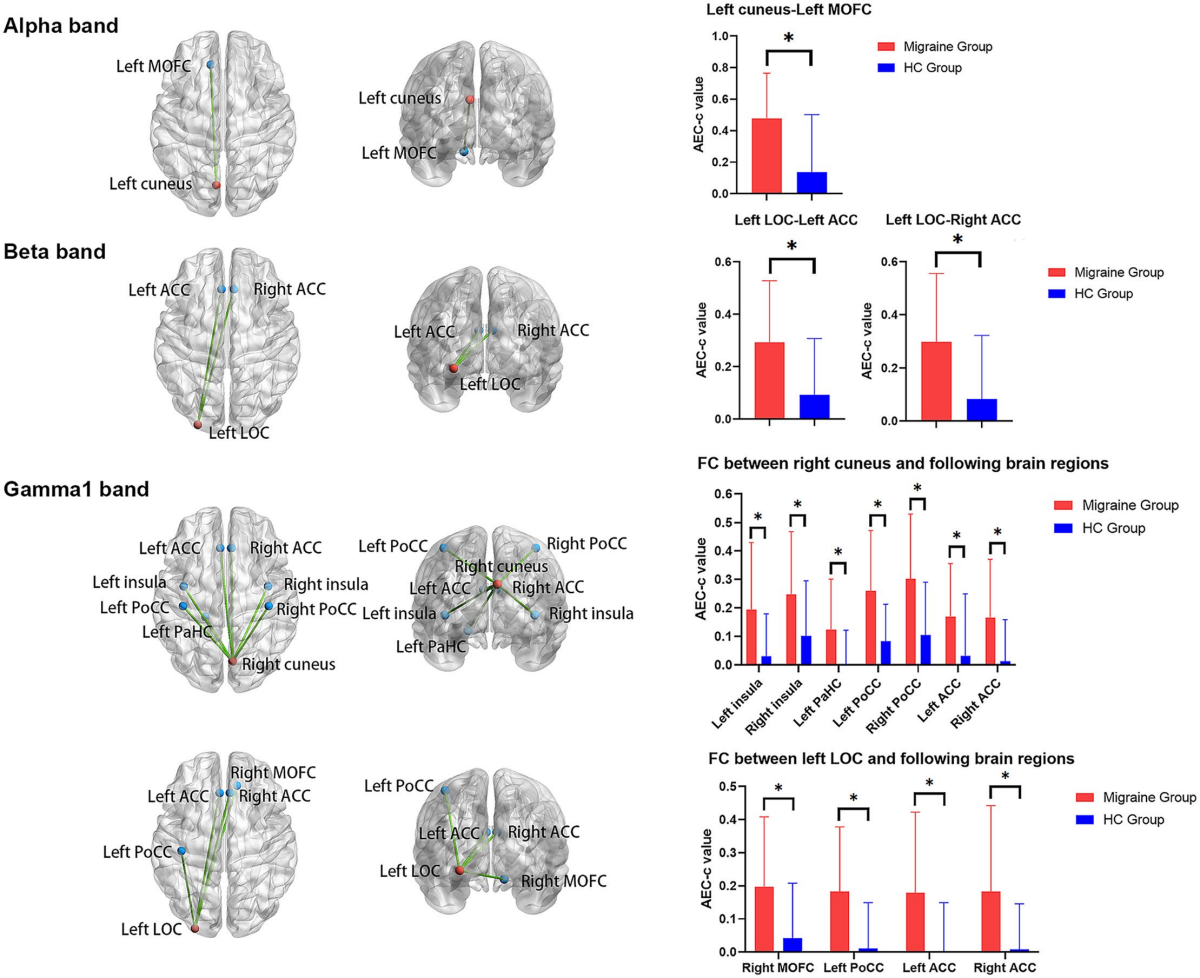
Significant FC differences between the migraine and HC groups. The FC maps are shown from the top and back views. Vision-related brain regions are represented by red balls, while pain-related brain regions are represented by blue balls. In the alpha band, the AEC-c value between the left cuneus and left MOFC was significantly increased in the migraine group. In the beta band, the AEC-c value between the left LOC and bilateral ACC was significantly increased in the migraine group. In the gamma1 band, the AEC-c values between the right cuneus and bilateral insula, left PaHC, bilateral PoCC, and bilateral ACC were significantly increased in the migraine group. In the gamma1 band, the AEC-c values between the left LOC and right MOFC, left PoCC, and bilateral ACC were significantly increased in the migraine group. MOFC, medial orbitofrontal cortex; LOC, lateral occipital cortex; ACC, anterior cingulate cortex; PaHC, parahippocampal cortex; PoCC, posterior central cortex. *p-values after correction were less than 0.05.

In the alpha (8–12 Hz) band, the migraine group had a significantly higher AEC-c value of the left cuneus to the left medial orbitofrontal cortex (*p_corrected_* = 0.028) compared with the HC group (Figure 2).

In the beta (15–29 Hz) band, the migraine group had significantly a higher AEC-c value of the left lateral occipital cortex to the left anterior cingulate cortex (*p_corrected_* = 0.049) and right anterior cingulate cortex (*p_corrected_* = 0.049) compared with the HC group (Figure 2).

In the gamma1 (30–59 Hz) band, the migraine group had a significantly higher AEC-c value of the right cuneus to the left insula (*p_corrected_* = 0.048), right insula (*p_corrected_* = 0.031), left parahippocampal cortex (*p_corrected_* = 0.048), left posterior central cortex (*p_corrected_* = 0.031), right posterior central cortex (*p_corrected_* = 0.031), left anterior cingulate cortex (*p_corrected_* = 0.031), and right anterior cingulate cortex (*p_corrected_* = 0.031) compared with the HC group (Figure 2). Additionally, the migraine group had a significantly higher AEC-c value of the left lateral occipital cortex to the right medial orbitofrontal cortex (*p_corrected_* = 0.033), left posterior central cortex (*p_corrected_* = 0.033), left anterior cingulate cortex (*p_corrected_* = 0.033), and right anterior cingulate cortex (*p_corrected_* = 0.042) compared with the HC group (Figure 2).

### Clinical associations

To shed light on clinical significance, we analyzed correlations between patients’ main clinical characteristics (headache history, attack frequency, and pain intensity) and abnormal MEG results (spectral power and FC).

Results of correlation analysis demonstrated that there were no significant correlations between migraine clinical characteristics (headache history, attack frequency, and pain intensity) and abnormal spectral power (*p_corrected_* > 0.05). There were no significant correlations between migraine clinical characteristics (headache history, attack frequency, and pain intensity) and abnormal AEC-c value (*p_corrected_* > 0.05).

## Discussion

This study investigated visual evoked magnetic fields among migraineurs from low to high frequency ranges, demonstrating that migraineurs have abnormal source spectral power activity and FC networks under visual stimulation in distinct frequency ranges.

Regarding spectral power, our study systematically analyzed visual stimuli-induced brain activation in the whole brain cortex between migraineurs and HCs at different frequency bands. Our data demonstrate that migraineurs have significantly decreased spectral power under visual stimuli in the orbitofrontal cortex, posterior cingulate cortex, temporal pole, and transverse temporal cortex, which are largely brain regions associated with pain processing. The orbitofrontal cortex is involved in pain processing and emotion and has been shown to release opioids in response to pain stimulation (48, 49). Some previous MEG studies have also found abnormalities in the prefrontal cortex of migraine patients during resting-state and negative emotional stimuli, suggesting that the prefrontal cortex may play a critical role in the pathogenesis of migraine (50, 51). Other studies have shown that the orbitofrontal cortex may be associated with the onset of chronic migraine and drug overuse headache, through emotional dysregulation (increasing anxiety/stress sensitivity), impaired pain modulation (exacerbating central sensitization), and disrupted reward evaluation/decision-making (52). In chronic migraine and medication-overuse headache, its dysfunction may impair the ability to assess the negative consequences of medication overuse, perpetuating compulsive drug-seeking behavior (53). The involvement of the posterior cingulate cortex in nociception has been demonstrated, with experimental evidence that pain deactivates this brain region (54). A resting-state MEG study has found that neural connectivity between the secondary somatosensory cortex and some pain-related cortices (i.e., the posterior cingulate cortex) were reduced by chronic pain in complex regional pain syndrome (55). A fMRI study has reported correlations between changes in the left posterior cingulate cortex and both the frequency and intensity of headaches (31). Our study revealed reduced activation in the posterior cingulate cortex of migraine patients, leading to impaired pain signal processing and multimodal information integration, thereby exacerbating pain hypersensitivity. The temporal lobe plays a pivotal role in migraine mechanisms, functioning as a sensory integration center (encompassing visual inputs), with its temporal pole specifically mediating ventral stream visual processing (56). Our results revealed decreased activation of the temporal pole in migraineurs under visual stimulation, which is consistent with previous research and suggests a potential association between aberrant temporal pole function and atypical processing of visual information in migraine patients. Cortese et al. (57) showed that, by enhancing excitability of the temporal pole using anodal transcranial direct current stimulation (tDCS), abnormal interictal visual information processing in migraineurs can be normalized. This observation leads to the hypothesis that the temporal pole could be a novel target for tDCS as a prophylactic treatment for migraine. Furthermore, our findings revealed decreased activation of the transverse temporal cortex in migraineurs under visual stimulation. The transverse temporal cortex is reported to be involved in the analgesic effect of acupuncture (58), suggesting a role in pain regulation. While existing neuroimaging studies have primarily employed MRI to assess structural alterations (e.g., cortical thickness) or functional changes (blood oxygen level dependent signals) in patients with migraine, the present study provides supplementary electrophysiological evidence by characterizing visual stimulation-evoked neural magnetic responses within these regions. Our findings that migraine patients exhibited abnormal activation of pain-related brain regions under visual stimulation further support the link between abnormal visual processing systems and migraine attacks.

To further explore the changes in neural networks under visual stimulation in migraineurs, we systematically analyzed FC between vision- and pain-related brain regions. Our results demonstrated that the migraineurs have significantly increased FC between vision-related brain regions (cuneus, lateral occipital cortex) and pain-related brain regions (orbitofrontal cortex, anterior cingulate cortex, insula, parahippocampal cortex, posterior central cortex) under visual stimulation in multiple frequency bands. Several studies have investigated alterations in FC among migraine patients using MEG. One of the first MEG connectivity study in migraine revealed enhanced slow-wave FC in frontal regions, while migraine with aura exhibited elevated theta-band connectivity in occipital areas (16). Another resting-state MEG study showed a lack of correlation between pain sensitivity in migraine patients and gamma oscillation in pain-related cortical regions (59). A recent study confirmed that the FC of migraineurs was lower than the HC group in the delta, alpha, and beta bands (60). These resting-state MEG studies have found that the abnormal functional connections of migraine are mainly located in the brain regions related to pain or vision. Two MEG studies have characterized dynamic FC patterns during task-state in patients with migraine. The MEG study by Ren et al. demonstrated significantly increased high-frequency band (250–1,000 Hz) connectivity in the somatosensory-frontal network during peripheral nerve stimulation among migraine patients during interictal periods (61). Additional MEG evidence revealed strengthened gamma-band effective connectivity from prefrontal to temporal cortices during negative emotional processing in migraine patients (51). These MEG findings indicate that in different task states, the FC of migraine patients across distinct brain regions is enhanced, and this change is frequency band-dependent. These research results are consistent with our finding that in the visual task state of migraine patients, the FC between vision-related and pain-related brain regions is enhanced in alpha, beta and gamma1 band. Visuo-nociceptive network synchrony may be involved in pain integration and play an important role in headache development and maintenance. Such findings could provide novel targets for migraine treatment. For example, Sava et al. demonstrated that repetitive transcranial magnetic stimulation used to experimentally inhibit the visual cortex reduced the supraorbital pain threshold and caused photophobia in healthy individuals (62). Based on these findings, the researchers proposed a functional link between the visual cortex and trigeminal nociceptive system. They also indicated that decreased activity in the visual cortex due to pathological reasons could contribute to the onset of headaches. These findings might help to understand the mechanism of aberrant visual phenomena in migraineurs, and might provide new insights into the pathophysiological mechanisms of migraine.

According to our results, most aberrant visual evoked magnetic signal parameters were in gamma-frequency oscillations (30–90 Hz). Gamma oscillations are generated within neural networks that include excitatory pyramidal cells and inhibitory gamma aminobutyric acid (GABA)-energic interneurons (63). Gamma oscillations have been shown to be associated with pain perception (64), attentional effect of pain (65), and subjective pain intensity (66). Previous studies have also suggested that properties of gamma oscillations are altered in migraineurs under resting-state and visual stimuli. A MEG study found that the spectral power in resting-state gamma-frequency oscillations among migraine patients was aberrant in the left frontal and left temporal regions (67). Similarly, two EEG studies have reported abnormal gamma-band power in migraineurs during visual stimulation (68, 69). Consistent with previous findings, we observed increased connectivity in gamma-band oscillations in response to visual stimulation in migraine. Increased connectivity in gamma-band oscillations in migraine could indicate a lack of gain control and less efficient processing in the brain (70, 71). As these oscillatory differences are known to be involved in cortical excitability and suppression of activity, altered gamma oscillations in migraineurs might reflect abnormal regulatory mechanisms of excitation and inhibition (71). Research at the molecular level has found a positive relationship between gamma oscillation peak frequency and GABA concentration in the motor cortex (72). Since migraine patients have reduced GABA levels, medications that enhance GABA may be effective against headache attacks (73). This result may contribute to understanding migraine pathophysiology and providing evidence for the treatment of migraine.

The current study is subject to several limitations. First, we did not find significant correlations between the abnormal MEG parameters (spectral power and FC) and related clinical characteristics (headache history, attack frequency, and pain intensity), which is in line with data presented in some MEG studies. This is likely due to limited number of subjects (11, 50). In future research, more subjects will be enrolled in the study. Second, we only included migraine without aura patients and did not analyze other subgroups. As the largest subgroup of migraine sufferers, many previous studies exploring visual cortical function have focused on migraine without aura patients (11, 18, 74). Further studies involving a larger number of migraine patients are needed to perform subgroup analysis to address possible differences in results between patients with migraine with and without aura, or between episodic migraine and chronic migraine. The last limitation is the use of a cross-sectional design. Longitudinal studies are necessary to determine if variation in brain activation or FC in migraine patients predisposes them to migraine or arise from recurrent migraine attacks. Prospective longitudinal studies could reveal aberrations in brain activation and FC that could serve as early biomarkers to predict alterations in migraine patterns.

## Conclusion

This study has demonstrated that migraine is associated with characteristic alterations in both spectral power and FC in response to visual stimuli during headache-free phases. Decreased spectral power in pain-processing brain regions and increased FC between vision- and pain-related brain regions are likely to play a role in migraine. These alterations are band-specific — especially in the gamma frequency band. These findings may contribute to better understanding the relationship between visual dysfunction and headache onset in migraine and provide valuable insights into the underlying pathophysiology.

## Data Availability

The raw data supporting the conclusions of this article will be made available by the authors, without undue reservation.

## Footnotes

^1^ https://surfer.nmr.mgh.harvard.edu/

^2^ https://www.bioladder.cn/web/#/chart/58

## References

[1] Dodick DW. Migraine. Lancet. (2018) 391:1315–30. doi: 10.1016/S0140-6736(18)30478-1, PMID: 29523342

[2] van Dongen RM Haan J. Symptoms related to the visual system in migraine. F1000Res. (2019) 8:F1000 Faculty Rev-1219. doi: 10.12688/f1000research.18768.1, PMID: 31448081 PMC6668047

[3] Russell MB Olesen J. A Nosographic analysis of the migraine Aura in a general population. Brain. (1996) 119:355–61. doi: 10.1093/brain/119.2.355, PMID: 8800932

[4] Leao AA. Further observations on the spreading depression of activity in the cerebral cortex. J Neurophysiol. (1947) 10:409–14. doi: 10.1152/jn.1947.10.6.409, PMID: 20268874

[5] Launer LJ Terwindt GM Ferrari MD. The prevalence and characteristics of migraine in a population-based cohort: the gem study. Neurology. (1999) 53:537–42. doi: 10.1212/wnl.53.3.537, PMID: 10449117

[6] Gopalakrishnan R Malan NS Mandava N Dunn EJ Nero N Burgess RC. Magnetoencephalography studies in migraine and headache disorders: a systematic review. Headache. (2025) 65:353–66. doi: 10.1111/head.14867, PMID: 39523760 PMC11794981

[7] Mehnert J Bader D Nolte G May A. Visual input drives increased occipital responsiveness and harmonized oscillations in multiple cortical areas in Migraineurs. Neuroimage Clin. (2019) 23:101815. doi: 10.1016/j.nicl.2019.101815, PMID: 30974326 PMC6458451

[8] Fong CY Law WHC Fahrenfort JJ Braithwaite JJ Mazaheri A. Attenuated alpha oscillation and Hyperresponsiveness reveals impaired perceptual learning in Migraineurs. J Headache Pain. (2022) 23:44. doi: 10.1186/s10194-022-01410-2, PMID: 35382735 PMC8981672

[9] Martin H Sanchez del Rio M de Silanes CL Alvarez-Linera J Hernandez JA Pareja JA. Photoreactivity of the occipital cortex measured by functional magnetic resonance imaging-blood oxygenation level dependent in migraine patients and healthy volunteers: pathophysiological implications. Headache. (2011) 51:1520–8. doi: 10.1111/j.1526-4610.2011.02013.x, PMID: 22082422

[10] Griebe M Flux F Wolf ME Hennerici MG Szabo K. Multimodal assessment of optokinetic visual stimulation response in migraine with Aura. Headache. (2014) 54:131–41. doi: 10.1111/head.12194, PMID: 23980899

[11] Zhou ZY Yu YW Wu D Liu HX Xiang J Wu T. Abnormality of visual Neuromagnetic activation in female Migraineurs without Aura between attacks. J Headache Pain. (2019) 20:7. doi: 10.1186/s10194-018-0957-9, PMID: 30651072 PMC6734467

[12] Bjork M Stovner LJ Hagen K Sand T. What initiates a migraine attack? Conclusions from four longitudinal studies of quantitative Eeg and steady-state visual-evoked potentials in Migraineurs. Acta Neurol Scand Suppl. (2011) 191:56–63. doi: 10.1111/j.1600-0404.2011.01545.x, PMID: 21711258

[13] Carvalho GF Mehnert J Basedau H Luedtke K May A. Brain processing of visual self-motion stimuli in patients with migraine: an Fmri study. Neurology. (2021) 97:e996–e1006. doi: 10.1212/WNL.0000000000012443, PMID: 34290130

[14] Fregnac Y. Dynamics of functional connectivity in visual cortical networks: an overview. J Physiol Paris. (1996) 90:113–39. doi: 10.1016/s0928-4257(97)81412-x, PMID: 9116656

[15] Pikor D Banaszek-Hurla N Drelichowska A Hurla M Dorszewska J Wolak T. Fmri insights into visual cortex dysfunction as a biomarker for migraine with Aura. Neurol Int. (2025) 17:15. doi: 10.3390/neurolint17020015, PMID: 39997646 PMC11858725

[16] Wu D Zhou Y Xiang J Tang L Liu H Huang S. Multi-frequency analysis of brain connectivity networks in Migraineurs: a magnetoencephalography study. J Headache Pain. (2016) 17:38. doi: 10.1186/s10194-016-0636-7, PMID: 27090418 PMC4835413

[17] Chamanzar A Haigh SM Grover P Behrmann M. Abnormalities in cortical pattern of coherence in migraine detected using ultra high-density Eeg. Brain Commun. (2021) 3:61. doi: 10.1093/braincomms/fcab061, PMID: 34258580 PMC8269966

[18] Wei HL Tian T Zhou GP Wang JJ Guo X Chen YC. Disrupted dynamic functional connectivity of the visual network in episodic patients with migraine without Aura. Neural Plast. (2022) 2022:9941832–10. doi: 10.1155/2022/9941832, PMID: 35035474 PMC8754605

[19] Xiong Z Tian C Zeng X Huang J Wang R. The relationship of functional connectivity of the sensorimotor and visual cortical networks between resting and task states. Front Neurosci. (2020) 14:592720. doi: 10.3389/fnins.2020.592720, PMID: 33510609 PMC7835730

[20] Boulloche N Denuelle M Payoux P Fabre N Trotter Y Geraud G. Photophobia in migraine: an Interictal pet study of cortical Hyperexcitability and its modulation by pain. J Neurol Neurosurg Psychiatry. (2010) 81:978–84. doi: 10.1136/jnnp.2009.190223, PMID: 20595138

[21] Posternack C Kupchak P Capriolo AI Katz BJ. Targeting the intrinsically photosensitive retinal ganglion cell to reduce headache pain and light sensitivity in migraine: a randomized double-blind trial. J Clin Neurosci. (2023) 113:22–31. doi: 10.1016/j.jocn.2023.04.015, PMID: 37150129

[22] Ninomiya T Sawamura H Inoue K Takada M. Segregated pathways carrying frontally derived top-down signals to visual areas Mt and V4 in macaques. J Neurosci. (2012) 32:6851–8. doi: 10.1523/JNEUROSCI.6295-11.2012, PMID: 22593054 PMC6622205

[23] Hodkinson DJ Veggeberg R Kucyi A van Dijk KR Wilcox SL Scrivani SJ. Cortico-cortical connections of primary sensory areas and associated symptoms in migraine. eNeuro. (2016) 3:ENEURO.0163–16.2016. doi: 10.1523/ENEURO.0163-16.2016, PMID: 28101529 PMC5239993

[24] Baier B de Haan B Mueller N Thoemke F Birklein F Dieterich M. Anatomical correlate of positive spontaneous visual phenomena: a voxelwise lesion study. Neurology. (2010) 74:218–22. doi: 10.1212/WNL.0b013e3181cb3e64 20083797

[25] Sulpizio V Committeri G Lambrey S Berthoz A Galati G. Selective role of lingual/Parahippocampal gyrus and Retrosplenial complex in spatial memory across viewpoint changes relative to the environmental reference frame. Behav Brain Res. (2013) 242:62–75. doi: 10.1016/j.bbr.2012.12.031, PMID: 23274842

[26] Skorobogatykh K van Hoogstraten WS Degan D Prischepa A Savitskaya A Ileen BM. Functional connectivity studies in migraine: what have we learned? J Headache Pain. (2019) 20:108. doi: 10.1186/s10194-019-1047-3, PMID: 31747874 PMC6868768

[27] Ke J Yu Y Zhang X Su Y Wang X Hu S. Functional alterations in the posterior insula and cerebellum in migraine without Aura: a resting-state Mri study. Front Behav Neurosci. (2020) 14:567588. doi: 10.3389/fnbeh.2020.567588, PMID: 33132860 PMC7573354

[28] Jin C Yuan K Zhao L Zhao L Yu D von Deneen KM. Structural and functional abnormalities in migraine patients without Aura. NMR Biomed. (2013) 26:58–64. doi: 10.1002/nbm.2819, PMID: 22674568

[29] Tso AR Trujillo A Guo CC Goadsby PJ Seeley WW. The anterior insula shows heightened Interictal intrinsic connectivity in migraine without Aura. Neurology. (2015) 84:1043–50. doi: 10.1212/WNL.0000000000001330, PMID: 25663219 PMC4352101

[30] Gu L Shu H Wang Y. Functional brain alterations in migraine patients: an activation likelihood estimation study. Neurol Res. (2023) 45:717–24. doi: 10.1080/01616412.2023.2199377, PMID: 37019685

[31] Amaral VCG Tukamoto G Kubo T Luiz RR Gasparetto E Vincent MB. Migraine improvement correlates with posterior cingulate cortical thickness reduction. Arq Neuropsiquiatr. (2018) 76:150–7. doi: 10.1590/0004-282x20180004, PMID: 29809228

[32] Garcia-Larrea L Peyron R. Pain matrices and neuropathic pain matrices: a review. Pain. (2013) 154:S29–43. doi: 10.1016/j.pain.2013.09.001, PMID: 24021862

[33] Schwedt TJ Chiang CC Chong CD Dodick DW. Functional Mri of migraine. Lancet Neurol. (2015) 14:81–91. doi: 10.1016/S1474-4422(14)70193-0, PMID: 25496899 PMC11318354

[34] Headache Classification Committee of the International Headache S. The international classification of headache disorders, 3rd edition (beta version). Cephalalgia. (2013) 33:629–808. doi: 10.1177/0333102413485658, PMID: 23771276

[35] Xiang J deGrauw X Korostenskaja M Korman AM O'Brien HL Kabbouche MA. Altered cortical activation in adolescents with acute migraine: a magnetoencephalography study. J Pain. (2013) 14:1553–63. doi: 10.1016/j.jpain.2013.04.009, PMID: 23792072 PMC3844550

[36] Tadel F Bock E Niso G Mosher JC Cousineau M Pantazis D. Meg/Eeg group analysis with brainstorm. Front Neurosci. (2019) 13:76. doi: 10.3389/fnins.2019.00076, PMID: 30804744 PMC6378958

[37] Florin E Baillet S. The brain's resting-state activity is shaped by synchronized cross-frequency coupling of neural oscillations. NeuroImage. (2015) 111:26–35. doi: 10.1016/j.neuroimage.2015.01.054, PMID: 25680519 PMC4387013

[38] Kanamori Y Shigeto H Hironaga N Hagiwara K Uehara T Chatani H. Minimum norm estimates in meg can delineate the onset of Interictal epileptic discharges: a comparison with Ecog findings. Neuroimage Clin. (2013) 2:663–9. doi: 10.1016/j.nicl.2013.04.008, PMID: 24179817 PMC3777706

[39] Hincapie AS Kujala J Mattout J Daligault S Delpuech C Mery D. Meg connectivity and power detections with minimum norm estimates require different regularization parameters. Comput Intell Neurosci. (2016) 2016:3979547. doi: 10.1155/2016/3979547, PMID: 27092179 PMC4820599

[40] Gramfort A Luessi M Larson E Engemann DA Strohmeier D Brodbeck C. Mne software for processing meg and Eeg data. NeuroImage. (2014) 86:446–60. doi: 10.1016/j.neuroimage.2013.10.027, PMID: 24161808 PMC3930851

[41] Desikan RS Segonne F Fischl B Quinn BT Dickerson BC Blacker D. An automated labeling system for subdividing the human cerebral cortex on Mri scans into Gyral based regions of interest. NeuroImage. (2006) 31:968–80. doi: 10.1016/j.neuroimage.2006.01.021, PMID: 16530430

[42] Niso G Tadel F Bock E Cousineau M Santos A Baillet S. Brainstorm pipeline analysis of resting-state data from the open meg archive. Front Neurosci. (2019) 13:284. doi: 10.3389/fnins.2019.00284, PMID: 31024228 PMC6461064

[43] Colclough GL Woolrich MW Tewarie PK Brookes MJ Quinn AJ Smith SM. How reliable are meg resting-state connectivity metrics? NeuroImage. (2016) 138:284–93. doi: 10.1016/j.neuroimage.2016.05.070, PMID: 27262239 PMC5056955

[44] Colclough GL Brookes MJ Smith SM Woolrich MW. A symmetric multivariate leakage correction for meg connectomes. NeuroImage. (2015) 117:439–48. doi: 10.1016/j.neuroimage.2015.03.071, PMID: 25862259 PMC4528074

[45] Brookes MJ Hale JR Zumer JM Stevenson CM Francis ST Barnes GR. Measuring functional connectivity using meg: methodology and comparison with Fcmri. NeuroImage. (2011) 56:1082–104. doi: 10.1016/j.neuroimage.2011.02.054, PMID: 21352925 PMC3224862

[46] Cheng CH Wang PN Mao HF Hsiao FJ. Subjective cognitive decline detected by the oscillatory connectivity in the default mode network: a magnetoencephalographic study. Aging. (2020) 12:3911–25. doi: 10.18632/aging.102859, PMID: 32100722 PMC7066903

[47] Godfrey M Singh KD. Measuring robust functional connectivity from resting-state meg using amplitude and entropy correlation across frequency bands and temporal scales. NeuroImage. (2021) 226:117551. doi: 10.1016/j.neuroimage.2020.117551, PMID: 33186722 PMC7836237

[48] Leknes S Tracey I. A common neurobiology for pain and pleasure. Nat Rev Neurosci. (2008) 9:314–20. doi: 10.1038/nrn2333, PMID: 18354400

[49] Biagianti B Grazzi L Gambini O Usai S Muffatti R Scarone S. Orbitofrontal dysfunction and medication overuse in patients with migraine. Headache. (2012) 52:1511–9. doi: 10.1111/j.1526-4610.2012.02277.x, PMID: 23145856

[50] Liu H Ge H Xiang J Miao A Tang L Wu T. Resting state brain activity in patients with migraine: a magnetoencephalography study. J Headache Pain. (2015) 16:525. doi: 10.1186/s10194-015-0525-5, PMID: 25968099 PMC4429423

[51] Ren J Yao Q Tian M Li F Chen Y Chen Q. Altered effective connectivity in migraine patients during emotional stimuli: a multi-frequency magnetoencephalography study. J Headache Pain. (2022) 23:6. doi: 10.1186/s10194-021-01379-4, PMID: 35032999 PMC8903691

[52] Gomez-Beldarrain M Carrasco M Bilbao A Garcia-Monco JC. Orbitofrontal dysfunction predicts poor prognosis in chronic migraine with medication overuse. J Headache Pain. (2011) 12:459–66. doi: 10.1007/s10194-011-0340-6, PMID: 21499917 PMC3139058

[53] Kenwood MM Souaiaia T Kovner R Fox AS French DA Oler JA. Gene expression in the primate orbitofrontal cortex related to anxious temperament. Proc Natl Acad Sci USA. (2023) 120:e2305775120. doi: 10.1073/pnas.2305775120, PMID: 38011550 PMC10710052

[54] Kong J Loggia ML Zyloney C Tu P LaViolette P Gollub RL. Exploring the brain in pain: activations, deactivations and their relation. Pain. (2010) 148:257–67. doi: 10.1016/j.pain.2009.11.008, PMID: 20005043 PMC2815185

[55] Iwatsuki K Hoshiyama M Yoshida A Uemura JI Hoshino A Morikawa I. Chronic pain-related cortical neural activity in patients with complex regional pain syndrome. IBRO Neurosci Rep. (2021) 10:208–15. doi: 10.1016/j.ibneur.2021.05.001, PMID: 34095892 PMC8167223

[56] Kravitz DJ Saleem KS Baker CI Ungerleider LG Mishkin M. The ventral visual pathway: an expanded neural framework for the processing of object quality. Trends Cogn Sci. (2013) 17:26–49. doi: 10.1016/j.tics.2012.10.011, PMID: 23265839 PMC3532569

[57] Cortese F Pierelli F Bove I Di Lorenzo C Evangelista M Perrotta A. Anodal transcranial direct current stimulation over the left temporal pole restores Normal visual evoked potential habituation in Interictal Migraineurs. J Headache Pain. (2017) 18:70. doi: 10.1186/s10194-017-0778-2, PMID: 28726157 PMC5517389

[58] Zhao J Guo LX Li HR Gou XY Liu XB Zhang Y. The effects of acupuncture therapy in migraine: an activation likelihood estimation Meta-analysis. Front Neurosci. (2022) 16:1097450. doi: 10.3389/fnins.2022.1097450, PMID: 36778899 PMC9911686

[59] Hsiao FJ Chen WT Liu HY Wang YF Chen SP Lai KL. Individual pain sensitivity is associated with resting-state cortical activities in healthy individuals but not in patients with migraine: a magnetoencephalography study. J Headache Pain. (2020) 21:133. doi: 10.1186/s10194-020-01200-8, PMID: 33198621 PMC7670775

[60] Zhang X Xu F Wu D Wang Y Chen Q Sun F. Altered Neuromagnetic activity in the default mode network in migraine and its subgroups (episodic migraine and chronic migraine). J Integr Neurosci. (2024) 23:19. doi: 10.31083/j.jin2301019, PMID: 38287847

[61] Ren J Xiang J Chen Y Li F Wu T Shi J. Abnormal functional connectivity under somatosensory stimulation in migraine: a multi-frequency magnetoencephalography study. J Headache Pain. (2019) 20:3. doi: 10.1186/s10194-019-0958-3, PMID: 30626318 PMC6734310

[62] Sava SL de Pasqua V Magis D Schoenen J. Effects of visual cortex activation on the nociceptive blink reflex in healthy subjects. PLoS One. (2014) 9:e100198. doi: 10.1371/journal.pone.0100198, PMID: 24936654 PMC4061134

[63] Whittington MA Traub RD Kopell N Ermentrout B Buhl EH. Inhibition-based rhythms: experimental and mathematical observations on network dynamics. Int J Psychophysiol. (2000) 38:315–36. doi: 10.1016/s0167-8760(00)00173-2, PMID: 11102670

[64] Gross J Schnitzler A Timmermann L Ploner M. Gamma oscillations in human primary somatosensory cortex reflect pain perception. PLoS Biol. (2007) 5:e133. doi: 10.1371/journal.pbio.0050133, PMID: 17456008 PMC1854914

[65] Hauck M Lorenz J Engel AK. Attention to painful stimulation enhances gamma-band activity and synchronization in human sensorimotor cortex. J Neurosci. (2007) 27:9270–7. doi: 10.1523/JNEUROSCI.2283-07.2007, PMID: 17728441 PMC6673131

[66] Zhang ZG Hu L Hung YS Mouraux A Iannetti GD. Gamma-band oscillations in the primary somatosensory cortex--a direct and obligatory correlate of subjective pain intensity. J Neurosci. (2012) 32:7429–38. doi: 10.1523/JNEUROSCI.5877-11.2012, PMID: 22649223 PMC6703598

[67] Li F Xiang J Wu T Zhu D Shi J. Abnormal resting-state brain activity in headache-free migraine patients: a magnetoencephalography study. Clin Neurophysiol. (2016) 127:2855–61. doi: 10.1016/j.clinph.2016.05.015, PMID: 27417062

[68] Coppola G Ambrosini A Di Clemente L Magis D Fumal A Gerard P. Interictal abnormalities of gamma band activity in visual evoked responses in migraine: an indication of Thalamocortical dysrhythmia? Cephalalgia. (2007) 27:1360–7. doi: 10.1111/j.1468-2982.2007.01466.x, PMID: 17986271

[69] Lisicki M D'Ostilio K Coppola G Nonis R Maertens de Noordhout A Parisi V. Headache related alterations of visual processing in migraine patients. J Pain. (2020) 21:593–602. doi: 10.1016/j.jpain.2019.08.017, PMID: 31586677

[70] Orekhova EV Stroganova TA Schneiderman JF Lundstrom S Riaz B Sarovic D. Neural gain control measured through cortical gamma oscillations is associated with sensory sensitivity. Hum Brain Mapp. (2019) 40:1583–93. doi: 10.1002/hbm.24469, PMID: 30549144 PMC6865508

[71] O'Hare L Tarasi L Asher JM Hibbard PB Romei V. Excitation-inhibition imbalance in migraine: from neurotransmitters to brain oscillations. Int J Mol Sci. (2023) 24:93. doi: 10.3390/ijms241210093, PMID: 37373244 PMC10299141

[72] Muthukumaraswamy SD Edden RA Jones DK Swettenham JB Singh KD. Resting Gaba concentration predicts peak gamma frequency and Fmri amplitude in response to visual stimulation in humans. Proc Natl Acad Sci USA. (2009) 106:8356–61. doi: 10.1073/pnas.0900728106, PMID: 19416820 PMC2688873

[73] Linde M Mulleners WM Chronicle EP McCrory DC. Valproate (Valproic acid or sodium valproate or a combination of the two) for the prophylaxis of episodic migraine in adults. Cochrane Database Syst Rev. (2013) 2013:611. doi: 10.1002/14651858.CD010611, PMID: 23797677 PMC10373438

[74] Wei HL Zhou X Chen YC Yu YS Guo X Zhou GP. Impaired intrinsic functional connectivity between the thalamus and visual cortex in migraine without Aura. J Headache Pain. (2019) 20:116. doi: 10.1186/s10194-019-1065-1, PMID: 31856703 PMC6924083

